# Learning mechanisms and outcomes of an interprofessional molecular pathology workshop for residents

**DOI:** 10.1016/j.acpath.2022.100056

**Published:** 2022-10-19

**Authors:** Malon Meeuwsen, Willeke A.M. Blokx, Marianne M. van den Hurk, Lia C.R.M.G. Fluit, Patricia J.T.A. Groenen

**Affiliations:** a Department for Research in Learning and Education, Radboudumc Health Academy, Nijmegen, the Netherlands; b Department of Pathology, Division of Laboratories, Pharmacy and Biomedical Genetics, University Medical Center Utrecht, Utrecht, the Netherlands; c Faculty of Social Sciences, Radboud University Nijmegen, Nijmegen, the Netherlands; d Dept. of Pathology, Radboud University Medical Centre Nijmegen, Nijmegen, the Netherlands

**Keywords:** Molecular pathology, Workshop, Interprofessional learning, Boundary crossing

## Abstract

The developments in targeted therapies and molecular pathology have changed the classification of tumors and precision oncology. Pathologists and clinical scientists in molecular pathology and oncologists have regular multidisciplinary meetings and are responsible for translating molecular results into an appropriate treatment plan. This requires expertise and skills to be effective team players. Interprofessional collaboration (IPC) is essential for professionals in medicine; however, learning opportunities in current resident training are limited. This narrative study explores the collaborative output and learning mechanisms of interprofessional learning (IPL) of residents of different disciplines in the Morphology & Molecular^PLUS^ workshop and its preparation. Topics that were discussed in the workshop were technologies for the detection of mutations, copy number variations, tumor mutational burden, and circulating tumor DNA (ctDNA) analysis in the context of differential diagnosis and precision oncology. Data were collected by analyzing pre- and post-workshop questionnaires and interviews. An interprofessional team of three residents of each hospital had to be formed by one of the residents, which was challenging as not all residents from a hospital knew each other. Residents reported to have got to know each other and have learned about each other's roles and perspectives. They gained knowledge of molecular pathology and the added value of IPC, in particular, for residents early in their training. Enabling meetings for medical residents of different disciplines to get acquainted was perceived as the most facilitating factor for IPL. Time constraints as the biggest barrier in daily practice. We recommend offering IPL activities as early as possible in residency programs.

## Introduction

Nowadays, molecular pathology has become an essential part of pathology and is an integral part of most pathology residency training programs in the Netherlands. Molecular results should be part of a state-of-the-art diagnosis^1^^,^^2^ for appropriate classification and advice for precision oncology in a specific tumor. Traditionally, the pathologist determines the diagnosis of the tumor based on the histological classification, immunohistochemical findings, and the diagnosis informs prognostic factors. The role of the clinical scientist in molecular pathology (CSMP), which is a recognized profession by the Dutch Pathology Society,^3^ reflects the developments in molecular pathology, especially in the field of (precision) oncology. A CSMP is a PhD or MD/PhD in molecular biology and/or molecular pathology and/or genetics, accomplished a post-doctoral training in this field, and subsequently has completed a 2-year training in molecular pathology. The CSMP is responsible for the molecular test and reporting the molecular test results in relation to tumor genetic concepts and molecular pathways.^2^ The pathologist needs to have a broad knowledge of molecular pathology and is responsible for integral reporting of the histopathological and molecular findings in the context of a tumor in an individual patient. Both the pathologists and the CSMPs have an advisory role for the main treating physician, the oncologist. The final translation into a treatment plan for the patient takes place in a multidisciplinary meeting. The medical professionals need the ability to continuously gain and integrate new scientific insights since developments in molecular pathology are enormous. In addition, professionals must be effective team players and understand each other's “language” in order to critically consider the new scientific developments and translate this into medical practice. Professionals in the field of oncology diagnostics are increasingly aware of the importance for interprofessional learning (IPL).^1^, ^2^, ^3^, ^4^

To achieve effective collaborative practices in cancer diagnostics, opportunities to learn and understand such practices are needed.^5^^,^^6^ This can be done through interventions that incorporate IPL. IPL supports learners to collaborate and communicate with professionals of other disciplines.^7^, ^8^, ^9^, ^10^ Eventually, IPL is a means to achieve interprofessional collaboration (IPC) in practice, because when learners are taught how to work collaboratively, they are able to participate in such a practice.^11^ There are multiple ways to look at learning processes in an IPL activity.^12^ One focuses on boundaries, crossing the boundaries, and the processes at the boundaries,^13^ which fits best to the situation in which pathologist, CSMP and oncologist work. The theory of boundary crossing is mainly about making connections between practices and preventing discontinuity when professionals move between these practices. Learning within this theory is perceived as interacting, moving across, or participating in different disciplines. The theory focuses on the processes that take place on and across the divisions between practices, i.e., boundaries. In the theory of boundary crossing that will be used to analyze learning in an IPL activity, four types of learning on and across the boundary are defined: role identity, coordination of meetings with different disciplines, perspective making & perspective taking, and transformation. Role identity is about knowing your own professional role and identity and about knowing the professional role and identity of the others. The learning potential resides in getting to know the contributions of the other trainees. Organization of meetings is about the contexts, procedures, and resources that are needed to ensure that disciplines can work collaboratively, and boundaries are crossed. The learning potential resides in knowing what is needed to move between the different practices. Perspective taking & perspective making is about being able to look at your own perspective through the eyes of the other (i.e., perspective taking) and learning to look differently at practices by learning about the perspective of the other and being able to take on the perspective of the others (i.e., perspective making). In this way, the differences between practices are being made explicit, and the professionals will be aware of it. The learning potential resides in being aware of the different perspectives of the different professionals. Transformation is about the changes in practices and learning processes that occur when new practices are being developed collaboratively. The learning potential resides in creating new practices between the professions and reconsider current practices and relations.

The aim of this study was to gain a deeper understanding of the collaborative output and the learning mechanisms of IPL of residents who participated in a molecular pathology workshop, called the Morphology & Molecular^PLUS^ (M&M^PLUS^) workshop that was based on IPL, in the context of boundary crossing. This M&M^PLUS^ workshop was a cased-based interprofessional workshop that was organized to encourage shared learning about cancer diagnostics, improve awareness of the importance of IPC, and translation and application of IPC into daily clinical practice. We identified enabling factors and barriers related to IPC in practice that can be useful to make the implementation of IPL in residency programs successful.

## Materials and methods

### Study design

The workshop M&M^PLUS^ was organized for self-formed teams from several hospitals. An experienced CSMP, also the program director of the CSMP residency in the Radboudumc and a dermatopathologist, also program director of the pathology residency in the Radboudumc designed the workshop and were the moderators on the day of the workshop. A treating medical oncologist was present in the workshop and acted as a counselor. The CSMP moderator coordinated the workshop.

As being the smallest group, the total number of CSMP residents determined the number of teams formed. Each CSMP resident was invited by the CSMP workshop coordinator to participate in the workshop and was asked to invite a pathology resident and clinical resident to join the team. The clinical residents were from medical oncology, pulmonary medicine, or hematology. The names and email addresses of the pathology and treating clinician residents were submitted to the CSMP workshop moderator. Four weeks after the initial invitation, the teams were informed about the assignment, which was to select, describe, explain, and present at least one illustrative patient case within the topic that was prearranged by the workshop moderators. They had to explain the case from the different team members perspectives and in the context of the learning objectives of the workshop. The learning objectives are shown in the Supplementary Information.

One week before the workshop, the residents were invited via e-mail to take part in the study about learning by the CSMP workshop coordinator. Measurements consisted of a pre- and post-workshop evaluation questionnaire for residents and semi-structured interviews with the residents and the moderators. The pre-workshop questionnaire measured the experiences of the participants during the preparation of the workshop. The post-workshop questionnaire was measured into three categories: learning outcomes, intentions, and suggestions for improvement. This last questionnaire was handed out at the end of the workshop day. The interviews focused on a deeper understanding of the opinions and beliefs of the participants and moderators about the IPL workshop and the transformative effect in practice. These interviews took about 30 min per person. The questions of pre- and post-workshop questionnaires are shown in Table 1. These questions were made by the workshop moderators and the experts of the Radboud health academy. The interview questions were generated and conducted by the experts of the Radboud health academy and are shown in the Supplementary Information.

**Table 1:** Questions asked in the pre- and post-workshop questionnaires.

Pre- or post-workshop	Questionnaire questions
Pre and post	I am: …. (name) O ... Pathology resident O ... Treating physician resident (medical oncology, pulmonary medicine, or otherwise) O ... Clinical scientist in molecular pathology resident I am in the … year of the … year residency.
Pre 1	Which actions did take you take to find the participants for the resident team that will take part in the workshop? 1a. Indicate what helped you with this, or what hindered you with this.
Pre 2	To what extent does this interprofessional assignment contribute to gaining more insight into you own professional contribution in the diagnostic process or in the treatment for patients? Are you becoming more aware of your role? Please explain your answer.
Post 1	Professional identity of the other/perspective taking: did the actual contact with your other colleagues during the workshop provide you with extra insights (beyond what you already learned during the preparation)? Please explain this.
Post 2	Professional identity: Did this workshop give you more insight into what the other profession does? If so; what impact does this have on your own work? If not; was this insight already known, and how did you obtain it before?
Post 3	What are the main educational points that you have learned from this workshop?
Post 4	Your contribution as a professional: What are the most important points that you were able to bring up in this workshop?
Post 5	Do you consider this interprofessional workshop useful for the general diagnostic process and/or treatment of the patient (i.e., not of the specific case that your team presented)? O ... Yes, because …. O ... Partly, because. … O ... No, because …..
Post 6	What specific intentions regarding the interprofessional approach in diagnosis/treatment will you take home? If you really do not have any intentions regarding the interprofessional approach, please make a specific other intention. 6a. Who or what will help you with this? 6b. When is your intention successful? 6c. Do you have any suggestions, ideas for other interventions (besides this workshop) to stimulate interprofessional learning?
Post 7	Are there particular points for improvement for your own training after following this workshop: what are they? What is needed to implement these?

### Ethical approval

Ethical approval from the Ethical Review Board of the Netherlands Association for Medical Education for this study was obtained (NERB no.: 2020.3.9, entitled: An exploration of the learning mechanisms in the Interprofessional learning workshops results for diagnostic Oncology in the light of boundary crossing).

The participants of the M&M^PLUS^ workshop about cancer diagnostics and treatment have agreed their participation in the interviews, in writing. In addition, they agreed to the usage of the data in further research.

### Method of data analysis

Template analysis was used to analyze the questionnaire answers and the interviews. Firstly, the transcripts were read and reread to familiarize with the data. Subsequently, the coding of the data was started. Sentences (or part of the sentences) in the transcripts and questionnaire answers were marked that corresponded to the same subject and were given a code. Sentences (or part of the sentences) that matched the subject were given the same code. The codes were derived from the theory of boundary crossing, the questions from the interview guide, and arose from close inspection of the data. After reading encoding the data, the codes were clustered into themes. According to the described research protocol^14^, the use of a priori themes within template analysis helped to focus on themes that needed to be incorporated into the analysis. The categories of the codebook and an example are described in Supplementary Tables 1 and 2, respectively. MM and CRMG discussed the data analyses iteratively; all inconsistencies in applications of the codebook were discussed and resolved toward consensus. After finalizing the codebook, two researchers independently coded one transcript and compared their codes to achieve the reliability of the codes. The inter-rater reliability was 0.81. Subsequently, transcriptions and questionnaires were coded and analyzed using the Atlas.ti (version 8.4.4) software package. The answers to the questionnaires and the transcripts were taken together as content. This content was analyzed in the context of the a priori themes; separate utterances of the text were extracted, classified, and gathered into these themes.

## Results

### The M&M^PLUS^ workshop

In the M&M^PLUS^ workshop, 22 residents participated: 10 CSMP residents, 8 pathology residents, and 4 medical oncology or pulmonary medicine residents. There were nine teams; in one of the teams, there was an additional CSMP resident because this resident only started three weeks before the workshop took place; in two teams, the same pathology resident and medical oncology resident worked with two different CSMP residents. In 4 of the 9 teams, no medical oncology and/or pulmonary medicine resident participated because the CSMP and pathology residents failed to engage a treating clinician resident.

The participating residents had different levels of training. In the Netherlands, the residency program for pathologists lasts five years and for treating clinicians six years. In the pathology residency program, a training in molecular pathology is integrated into multiple themes. The residency program for CSMP lasts two years; for this residency, a Ph.D. or MD/Ph.D. in molecular biology and/or molecular pathology and/or genetics is mandatory and should be preferably accomplished a post-doctoral training in this field.^14^ In the workshop, 4 out of 10 CSMP residents were in year 1 of their training, 4 out of 8 pathology residents were in year 1 or 2 of their residency program, while 3 out of the 4 treating clinician residents were in later years of their training. The detailed information about the level of training as well as the number of residents per discipline who participated in the questionnaires and in the interviews is presented in Supplementary Table 3.

The teams prepared a presentation about a topic, *e.g*., a specific tumor type and/or technology and the clinical consequences for the patient. The participants had two months, but including the summer holidays, to prepare their 20 min presentation. On the day of the workshop, diverse and interesting cases were presented, as illustrated in Table 2, to the participants and moderators. After each case presentation, there was ample time for discussion about technological and medical aspects, about potentials, and pitfalls related to the case and/or the assignment, and the key learning points were demarcated. Also, attention was given to the information that was provided in the molecular pathology reports. At the end of workshop day, there was a final discussion focused on the learning points, the role of the professional, and the collaboration with other professionals. This discussion and the post-workshop questionnaires that were submitted revealed data about learning new technological and medical insights as well as about awareness of the importance of collaboration by the participants.

**Table 2:** Technical and medical learning points of the M&M^PLUS^ workshop

Team	Case	Key learning points
Team 1	Lung cancer and Tumor mutational burden (TMB)	The relevance of mutations/translocation for diagnosis of adenocarcinoma or unknown primary, the value and prerequisites of robust testing to determine tumor mutation burden and mutational signatures (smoking) in view of treatment options.
Team 2	Lung cancer and TMB	Pathology diagnosis of lung cancers; essential molecular parameters for the diagnosis of neuroendocrine carcinoma, the relevance of synoptic molecular reporting and understanding of these reports by the pathologist and the clinical doctors; discussion in a Molecular Tumor Board.
Team 3	Lung cancer and circulating tumor DNA (ctDNA)	Non-small cell lung cancer, non-invasive molecular analysis of ctDNA facilitates early detection of resistance mutations (EGFR p.T790M and p.C797S); the ins and outs of the used method.
Team 4	Colorectal cancer	Lynch syndrome with endometrioid adenocarcinoma, a mismatch repair deficient carcinoma: a case with a *PMS2* germline mutation and *MLH1* promoter hypermethylation
Team 5	Glioma	Diffuse astrocytoma (WHO grade 2) without IDH1/2 mutation, without co-deletion of 1p/19q, however, with a *TERT* promotor mutation: consequences for diagnosis and treatment
Team 6	Lymphoma	Difficult diagnosis; lymphoplasmacytic lymphoma, follicular lymphoma, or extranodal marginal zone lymphoma; pro's and con's of break-apart or fusion probes for FISH, and an illustration of the value of accurate reporting and interprofessional collaboration
Team 7	Myeloid	From essential thrombocytopenia to acute myeloid leukemia, clonal evolution and insight in copy number variations (CNV) and copy neutral loss of heterozygosity using array technology
Team 8	Melanocytic lesion	The relevance of specific mutations and CNV detection in the diagnosis of cutaneous intermediate melanocytic lesions.
Team 9	Urothelial carcinoma	A dedifferentiated urothelial carcinoma in the bladder in a patient with prostate cancer, with identical *TP53* and *PTEN* mutations; the importance of synoptic reporting of pathology data and interprofessional collaboration in view of diagnosis and treatment

The pathology residents in year 1 or 2 of the residency program indicated that they had become more aware of the possibilities of the molecular diagnostics and the consequences. The pathology residents in year 3, 4, or 5 of the residency program did not mention this aspect, but specifically noticed the importance of good communication and collaboration between the professionals. A pathologist in the 5th year of the residency stressed the importance of illustrating the shortcomings of a technology, hence explaining an uncertain diagnosis of a patient, in multidisciplinary meetings. Another pathology resident in year 4 of the training indicated that the workshop challenged him to practice pathology from a more holistic view. The treating clinician residents all indicated the importance of learning about possibilities of pathology and the molecular diagnostic technologies and the interpretation of the results. One treating clinician resident in the 4th year of the residency program noticed that some of the features in a pathology report had not been fully clear before, while these can be clinically relevant. The pathology and treating clinician residents became more aware that different molecular assays were used in the different molecular pathology laboratories in the Netherlands. The CSMP residents in the first year of training described that they obtained more insight into the multitude and the complexity of molecular tests and also learned about histology, while residents later in their training already had these insights. But these second-year CSMP residents learned more about specific applications or interpretations of (molecular) pathology tests results. First-year CSMP residents also better appreciated the level of knowledge domain and the responsibilities of a CSMP as well as the importance of communication with pathologist and treating clinicians. Year 2 CSMP indicated to have participated in multidisciplinary tumor boards and/or meetings and were already aware of this, although they also stress the importance of close IPC.

### Learning processes in the workshop

By discussing cases that were presented with their group members, residents indicated that they learned about each other's roles and perspectives: *“you had to discuss a case from every point of view. In this way, everyone's tasks became clear.’’* Also, when the interprofessional teams prepared the presentation for the workshop, they learned to coordinate and collaborate with each other. Additionally, they learned to translate the knowledge about their own field more clearly in a way that made it easier to understand by other disciplines. The interviews with the participants revealed that the possibility of “creating new practices” was not explicitly discussed in the workshop.

### Learning outcomes and behavioral changes

In the analysis of the categorical content from the pre- and post-workshop questionnaires and the transcripts of the interviews, which were processed, combined, and summarized, we recognized learning on and across the boundaries of different professionals. Exemplary quotes categorized within the types of learning mechanisms of boundary crossing; role identity, organization of meetings of different disciplines, perspective making & perspective taking, and transformation are presented in Table 3. Residents learned most about the professional roles and perspectives of the other disciplines and the boundaries between different fields, which corresponds to role identity and perspective making, both features of the boundary cross. Because of the new insights into each other's roles, participants said they could provide the targeted information to the other disciplines. As a result, the participants learned who to ask best for specific for information and were able to ask more specific questions.

**Table 3:** Boundary crossing learning: definitions and illustrative quotes from the interviews.

Learning mechanism	Definition	Illustrative quote
Role identity	Getting to know your own professional role and identity and the professional role and identity of the other residents. The learning potential resides in getting to know the contributions of the other residents.	*“Everyone presented their part in the diagnostic process, where it starts, and what is covered by their role and where they need input or expertise of our partners. That's how we got to know each other's roles.”“What I learned about my own role, is what kind of questions I should be able to answer and what information they require of me.”*
Organization of meetings for different disciplines	Learning about the contexts that are needed to ensure that disciplines can work collaboratively. The learning potential resides in knowing what is needed to move between the different practices.	*“I was thinking ‘wait a minute’, how do I find exactly who I need for this case?”“The moderators of the workshop certainly emphasized to consult with each other as much as possible. At least, that was the main message of the workshop for me.”“Education can bring disciplines together”*
Perspective taking & perspective making	Being able to look at your own perspective through the eyes of the other residents (i.e., perspective taking) and being able to look differently at oncology diagnostics by learning about the perspective of the other residents and being able to take on the perspective of the others (i.e., perspective making). The learning potential resides in being aware of the different perspectives of a clinical molecular pathologist, a pathologist, and a medical oncologist.	*“Right now, in practice, I sometimes think about how to formulate certain results in a report, so the other discipline also understands the report.”“The workshop challenged me to look differently at the daily practice. I realized there is another side to it and became aware of the perspective of the other, for example, on a gene mutation.”*
Transformation	The changes in practices and learning processes that occur when new practices are being developed collaboratively. The learning potential resides in creating new practices between the professions.	*“There was not really any emphasis on how we could work together in a different or more efficient way after finishing the workshop.”“The workshop has led in concrete terms to a discussion with the pathology residents and we are planning to engage our trainers to better integrate molecular diagnostics into our training program. But there is no concrete change yet.”*

Most residents were also able to take on each other's perspective (i.e., perspective taking). The participants learned new technical and medical insights about their own and about the other disciplines. Because participants had an overview of the entire diagnostic workflow, they became more aware of the importance of collaboration. Although they reported to have gained insight into the activities of the other disciplines and into the importance of their activities for the other disciplines, the participants did not report specifically on learning about looking at their own perspective through the eyes of the other (i.e., perspective making). Regarding coordination of meetings with different disciplines, residents knew what is needed to move between practices which are multidisciplinary meetings or department meetings and education.

When looking at transformation, all participants learned new technical and medical insights about their own and other disciplines. Because participants got an overview of the entire diagnostic workflow, they became more aware of the importance of collaboration. Also, the participants experienced more intensive IPC after participating in the workshop, because they got to know each other through the workshop. The interviews, which took place eight months later than the workshop, revealed that a couple of new practices were created: visiting each other more often and reconsidering current practices and relation, and they communicated more clearly also with respect to the patient reports. Some of the participants noted that they had become involved in educational activities for the other disciplines.

The interviews also revealed that the biggest barrier to collaboration in daily practice perceived by all participants was a lack of time. Additionally, both the attitude of colleagues and of residents themselves can hinder collaboration. For example, *“It is a pitfall that everyone thinks ‘I will wait until the other person e-mails’ and then nobody takes the initiative.”*

Both moderators were enthusiastic about facilitating the workshop. They learned most about molecular pathology and the procedures in other hospitals. Additionally, they reported positive changes in the behavior of the residents after participating in the workshop, like educating other disciplines and knowing each other's roles and perspectives. However, they also mentioned that *“the residents consider a technical or medical feature more important than paying attention to the cooperation.”*

## Discussion

The M&M^PLUS^ workshop was organized for self-formed multidisciplinary teams of different Dutch hospitals. By incorporating the treating physician, this workshop was an extension of a former “M&M workshop” for teams consisting of residents in CSMP and pathology only. The involvement of a treating clinician resident was considered as a hurdle as the majority of CSMP residents did not know the residents in medical oncology, pulmonary medicine, or hematology, while most of them knew the pathology residents. Unfortunately, nearly half of the teams did not manage to involve a resident-treating clinician in this workshop. They needed advice from their supervisor(s) to get in touch with the residents of another discipline. In fact, a formal assignment like this workshop was necessary to allow them to meet one another.

The M&M^PLUS^ workshop, in which several disciplines participated, contributed to a better understanding of the different technologies, their potentials and pitfalls and to better appreciation of the different workup procedures in the evaluation of the histology and the supplementary technologies for certain tumor types in the different hospitals. The importance of proper understanding a molecular report by the pathology and the treating clinician residents became clear; the residents became more aware of the specific features in the molecular report and their importance. Also, they became more conscious that there are in-between hospital variations in gene panels and assays used.^3^^,^^15^, ^16^, ^17^ A few laboratories already used synoptic reporting (i.e., uniform) of molecular pathology data that has been introduced in the Netherlands, similarly to the synoptic reporting of pathology that was introduced more than a decade ago.^18^^,^^19^ Minor differences in data description in the narrative part of the synoptic pathology reports were noted that could potentially be important for an accurate understanding of challenging diagnostic cases. Also, the importance of the clinical context and decision-making was well represented in the workshop.

Besides learning and exchange of relevant technical and medical information, from multiple perspectives, the workshop for cancer diagnostics resulted in contact between residents who did not knew each other well before and a deeper understanding of IPL and beneficial learning experiences. Our study indicated that learning through the mechanisms of boundary crossing was effective to learn about IPL and that residents early in their residency training learned the most. After participating in the workshop, participants gained more knowledge regarding IPC. The data of our study suggested that participants mostly gained knowledge about subject matter and social contacts. The interviews demonstrated that the learned competencies led to an adaptation of behavior in practice, i.e., “transformation”: more contacts, more reaching out to each other, taking more awareness of the other profession when doing their own work (like when making patient reports). For some participants, it also yielded something in addition, since they became involved in educational activities for the other disciplines.

Most likely, the transformation of practices, in general, will hardly be achievable after participating in a single IPL activity since it requires changes in behavior and/or organization. Repetition of either the workshop or alternative interdisciplinary meetings would be needed, as the participants indicated. The most facilitating factor for IPC according to the residents was organizing educational meetings to create meetings with residents from different disciplines. Being close to each other or working in the same room/floor helps to come more into contact. Residents perceived a high workload, poor relationships between different disciplines, unsupportive supervisors, not taking initiative, a lack of communication, not working close to each other, and no planned moments of contact as barriers for IPC. The biggest barrier, however, was a lack of time.

A strength of this study was that multiple perspectives were included: residents and moderators from different academic hospitals in the Netherlands shared their experiences and thoughts. Another strength is that the study was set up using theoretical concepts that helped participants learn more about IPC. The evaluation of a single workshop with a small group of participants could be considered as a weakness of our study. In addition, only perceptions were described and studied; there was no observation of what actually happened in practice. Also, our study was limited by a possible selection bias in the results, as the residents participated voluntarily in the interviews. One might envisage the eight-month interval between the workshop and the interviews as a limitation, as the residents could have forgotten what exactly happened in the workshop. On the other hand, eight-month is needed and sufficient to introduce the changes that the participants indicated.

Although there are meetings, congresses, and workshops^4^ where pathologists, molecular biologists, and professionals from other fields meet, IPL and IPC have limited representation in residency programs. We recommend to repeatedly plan IPL meetings and activities (assignments), so that the residents of different programs learn how to create new practices and work more efficiently together. Our study showed, as one could have expected, that residents early in their residency had the most benefit from the workshop and could make a behavioral change. We, therefore, strongly advise to start integrating IPL early in the residency programs. Preparation of and participation in (molecular) tumor boards with residents from different disciplines most likely is also an effective intervention.^20^

In summary: to the best of our knowledge, this is the first study that focuses on the learning mechanisms of boundary crossing^13^ in relation to an IPL activity. With regard to IPL and IPC, our study showed that such a workshop encouraged meeting of residents of different disciplines and shared learning. Effective IPC needed a formal assignment to allow them to meet each other. This workshop in cancer diagnostics was most valuable early during residency since residents came into contact with each other and it increased knowledge and improved IPC between residents in the remainder of their residency. Meetings and collaborative assignments for medical residents of different disciplines improved awareness of the importance of IPC.

## Author contributions

PJTAG organized the interprofessional workshop; PJTAG and WAMB were faculty of the workshop; PJTAG, WAMB, and CRMGF formulated the pre- and post-workshop questionnaire. MM, PJTAG, and CRMGF designed the study; MM and CRMGF analyzed the data. All authors had access to the study data at the time of the analysis. The original questionnaires are stored at the Department of Pathology (PJTAG), the evaluation data files of the questionnaires and the interviews are stored at the Radboud Health Academy (CRMGF). CRMGF, MMH, and PG supervised the reported data. MM, WAMB, and PJTAG wrote the manuscript. All co-authors declared that they had access to the data in the study and take responsibility for the integrity of the data and the accuracy of the data analysis. All co-authors critically revised the manuscript for important intellectual content.

## Declaration of competing interest

The authors report no conflicts of interest, no disclosures. The authors alone are responsible for the content and writing of this article.
