# Supplementary material for: Learning mechanisms and outcomes of an interprofessional molecular pathology workshop for residents
Source: Acad Pathol. 2022 Oct 19;9(1):100056. doi: 10.1016/j.acpath.2022.100056 (PMC9587361; doi:10.1016/j.acpath.2022.100056)
Supplement: Multimedia component 1 [file mmc1.docx]

Supplementary Information

Learning mechanisms and outcomes of an interprofessional molecular pathology workshop for residents.

M. Meeuwsen *et al.*

Learning objectives of the M&M^plus^ workshop:

**For pathology residents**

The pathology resident….

- is able to explain the results of the molecular pathology patient report

- is aware of the different molecular tests and the applications, sensitivity and turn-around-time

- can interpret the sensitivity and limitations of a molecular test in a patient report

-is able to explain the consequences of the molecular results in the context of (differential) diagnosis and the prediction of therapy response

**For CSMP residents**

The CSMP resident is …..

- able to evaluate the importance of a diagnostic request by the pathologist

- able to recognize and explain unexpected findings that not entirely fit the diagnosis or request

- aware of the general pathology tests that are possible using diagnostic pathology specimen, in the context of (differential) diagnosis and prediction of therapy response

- able to recognize tumor cells and tumor infiltrative lymphocytes in tumors

- able to explain the consequences of the molecular results in the context of prediction of therapy response

**For treating clinician residents**

The treating clinician resident….

- can reason the importance of a diagnostic request for molecular pathology with the use of the available diagnostic specimen

- is able to explain the consequences of the molecular results in the context of prediction of therapy response.

- can interpret the sensitivity and limitations of a molecular test in a patient report

- understands that there are different molecular tests for different applications and is informed about the turn-around-times.

INTERVIEW GUIDELINE:

# Interview guide for interviewing trainees

**Introduction**

- Introducing myself
- Did you read the information letter in de e-mail?
- Do you have questions?

**Goal of the interview**

- This interview is about your experience of the workshop en de impact of the workshop on your workactivities.

**Ethical**

- This interview is confidential and will be anonymized.
- The interview will be recorded by audio-recorder. The recording will be deleted after transcription of the interview. Passages containing traceable information will be anonymized.
- You can always end the interview when you want to. In that case, the information up to that point is not included.

**Start recording**

**Introducing questions**

- What is your specialty?
- How did you experience participating in the workshop?

**Vision of the workshop**

- Did you find the workshop valuable?
  - If so, why?
- Do you think this workshop contributes to interprofessional learning? Why yes/no?
- What do you think are facilitating factors to promote interaction and learning between the trainees of different disciplines?
- What do you think are facilitating factors to promote interaction and learning between the trainees of different disciplines?
- What do you think are barriers to promote interaction and learning between the trainees of different disciplines?

**Content of the workshop**

- Which aspects of the workshop helped to promote interprofessional learning?
- Which aspects of the workshop did not help to promote interprofessional learning?

In this research we will investigate which ways of learning from the theory of boundary crossing occur. There are four ways of learning: role identity, organization of meetings with different disciplines, perspective making & perspective taking and transformation. Role identity is about knowing your own professional role and identity and about knowing the professional role and identity of the others. Did this occur in the workshop?

Organization of meetings, is about the contexts, procedures and resources that are needed to ensure that disciplines can work collaboratively, and boundaries are crossed. Did this occur in the workshop?

Perspective taking & perspective making is about being able to look at your own perspective through the eyes of the other (i.e. perspective taking) and learning to look differently at practices by learning about the perspective of the other and being able to take on the perspective of the others (i.e. perspective making). In this way, the differences between practices are being made explicit and the professionals will be aware of it. Did this occur in the workshop?

Transformation is about the changes in practices and learning processes that occur when new practices are being developed collaboratively. Did this occur in the workshop?

**Outcome of the workshop**

- Do you recognize the learning mechanism of role identity while working after participating in the workshop?
  - If so, can you specify this?
  - If not, how should this be expressed?
  - What could stimulate learning about one’s own professional role and identity and the other in practice?
  - What could obstruct learning about one’s own professional role and identity and the other in practice?
- Do you recognize the learning mechanism of organization of meetings while working after participating in the workshop?
  - If so, can you specify this?
  - If not, how should this be expressed?
  - What could stimulate creating contexts to learn from each other in practice?
  - What could obstruct creating contexts to learn from each other in practice?
- Do you recognize the learning mechanism of perspective making & perspective taking while working after participating in the workshop?
  - If so, can you specify this?
  - If not, how should this be expressed?
  - What would be beneficial in practice for the application of perspective taking and making?
  - What would hinder for the application of perspective taking and making in practice?
- Do you recognize the learning mechanism of transformation while working after participating in the workshop?
  - If so, can you specify this?
  - If not, how should this be expressed?
  - What would be beneficial for realizing changes with regard to collaboration in daily practice?
  - What would hinder the realization of changes with regard to collaboration in daily practice?
- What do you think is the impact of the workshop on oncological diagnostics?
- Do you think there are opportunities to apply what you have learned about IPL in daily practice?
- Do you think there are threats to apply what you have learned about IPL in daily practice?

**Ending**

- Are there any things you thought we would talk about beforehand, but I haven’t asked you anything about?
- What else do you want to say?

**Stop recording**

# Interview guide for interviewing moderators

**Introduction**

- Introducing myself
- Did you read the information letter in de e-mail?
- Do you have questions?

**Goal of the interview**

- This interview is about your experience of the workshop en de impact of the workshop on your workactivities.

**Ethical**

- This interview is confidential and will be anonymized.
- The interview will be recorded by audio-recorder. The recording will be deleted after transcription of the interview. Passages containing traceable information will be anonymized.
- You can always end the interview when you want to. In that case, the information up to that point is not included.

**Start recording**

**Introducing questions**

- What did you think about facilitating the workshop?
- Can you explain what interprofessional learning means?

**Vision of the workshop**

- Did you find the workshop valuable?
  - If so, why?
- Do you think this workshop contributes to interprofessional learning? Why yes/no?
- What do you think are facilitating factors to promote interaction and learning between the trainees of different disciplines?
- What do you think are facilitating factors to promote interaction and learning between the trainees of different disciplines?
- What do you think are barriers to promote interaction and learning between the trainees of different disciplines?

**Content of the workshop**

- Which aspects of the workshop helped to promote interprofessional learning?
- Which aspects of the workshop did not help to promote interprofessional learning?

In this research we will investigate which ways of learning from the theory of boundary crossing occur. There are four ways of learning: role identity, organization of meetings with different disciplines, perspective making & perspective taking and transformation. Role identity is about knowing your own professional role and identity and about knowing the professional role and identity of the others. Did this occur in the workshop?

Organization of meetings, is about the contexts, procedures and resources that are needed to ensure that disciplines can work collaboratively, and boundaries are crossed. Did this occur in the workshop?

Perspective taking & perspective making is about being able to look at your own perspective through the eyes of the other (i.e. perspective taking) and learning to look differently at practices by learning about the perspective of the other and being able to take on the perspective of the others (i.e. perspective making). In this way, the differences between practices are being made explicit and the professionals will be aware of it. Did this occur in the workshop?

Transformation is about the changes in practices and learning processes that occur when new practices are being developed collaboratively. Did this occur in the workshop?

**Outcome of the workshop**

- Do you recognize the learning mechanism of role identity while working after participating in the workshop?
  - If so, can you specify this?
  - If not, how should this be expressed?
  - What could stimulate learning about one’s own professional role and identity and the other in practice?
  - What could obstruct learning about one’s own professional role and identity and the other in practice?
- Do you recognize the learning mechanism of organization of meetings while working after participating in the workshop?
  - If so, can you specify this?
  - If not, how should this be expressed?
  - What could stimulate creating contexts to learn from each other in practice?
  - What could obstruct creating contexts to learn from each other in practice?
- Do you recognize the learning mechanism of perspective making & perspective taking while working after participating in the workshop?
  - If so, can you specify this?
  - If not, how should this be expressed?
  - What would be beneficial in practice for the application of perspective taking and making?
  - What would hinder for the application of perspective taking and making in practice?
- Do you recognize the learning mechanism of transformation while working after participating in the workshop?
  - If so, can you specify this?
  - If not, how should this be expressed?
  - What would be beneficial for realizing changes with regard to collaboration in daily practice?
  - What would hinder the realization of changes with regard to collaboration in daily practice?
- What do you think is the impact of the workshop on oncological diagnostics?
- Do you think there are opportunities for applying what the participants have learned about interprofessional learning in daily practice?
- Do you think there are threats for applying what the participants have learned about interprofessional learning in daily practice?

**Ending**

- Are there any things you thought we would talk about beforehand, but I haven’t asked you anything about?
- What else do you want to say?

**Stop recording**
