# Supplementary material for: Learning mechanisms and outcomes of an interprofessional molecular pathology workshop for residents
Source: Acad Pathol. 2022 Oct 19;9(1):100056. doi: 10.1016/j.acpath.2022.100056 (PMC9587361; doi:10.1016/j.acpath.2022.100056)
Supplement: Multimedia component 2 [file mmc2.docx]

**Supplementary Table 1.** The eight categories (1 to 8) of the codebook and corresponding subcategories (1.1 to 4.10 and 8.1 to 8.5).

| 1. Before the workshop | 2. During the workshop | 3. After the workshop | 4. Influencing factors | 5. Outcomes for moderators | | 6. Appreciation of the workshop | | 7. Importance of inter-professional collaboration | 8. Suggestions |
| --- | --- | --- | --- | --- | --- | --- | --- | --- | --- |
| - 1. Contact   2. Collaboration | - 1. Contact   2. Collaboration   3. Way of learning      1. Role identity      2. Organization of meetings      3. Perspective taking and making      4. Transformation | - 1. Contact   2. Subject matter   3. Perspective taking and making   4. Role identity   5. Education   6. Attitude   7. Organization of meetings   8. Collaboration   9. Transformation   10. No change | - 1. Contact   2. Education   3. Time   4. Workload   5. Crisis   6. Colleagues   7. Location   8. Attitude   9. Planning and organization   10. Recurrence |  |  | |  | | - 1. Collaboration   2. Contact   3. Attitude   4. Education   5. Workshop |
