# Supplementary material for: Learning mechanisms and outcomes of an interprofessional molecular pathology workshop for residents
Source: Acad Pathol. 2022 Oct 19;9(1):100056. doi: 10.1016/j.acpath.2022.100056 (PMC9587361; doi:10.1016/j.acpath.2022.100056)
Supplement: Multimedia component 3 [file mmc3.docx]

**Supplementary Table 2.** Example of coding the data.

| R: did you learn something about your own role? | Light Grey = 2.3.1 role identity  Dark Grey = 2.3.3. perspective taking and making |
| --- | --- |
| I: yes |  |
| R: What exactly did you learn? |  |
| I: what kind of questions you need to be able to answer and what information they require from you. |  |
| R: are you able to look through the eyes of the other at your own discipline? |  |
| I: Yes, but it is hard to say. You sometimes have tunnel vision when you’re working within your own discipline. And that is why these kinds of moments are very helpful to meet each other, because then you are reminded of the other’s profession. When working together, you think: ‘’ah yes, there’s another side to the work we do’’. |  |

R = researcher

I = interviewer
