# Supplementary material for: Learning mechanisms and outcomes of an interprofessional molecular pathology workshop for residents
Source: Acad Pathol. 2022 Oct 19;9(1):100056. doi: 10.1016/j.acpath.2022.100056 (PMC9587361; doi:10.1016/j.acpath.2022.100056)
Supplement: Multimedia component 4 [file mmc4.docx]

**Supplementary Table 3.** The length of the different residency programs, the number of residents per discipline per level of training and number per discipline that completed the pre- and post-workshop questionnaires and that participated in the interviews.

|  | Clinical scientist in molecular pathology | Pathologist | Treating physician (medical oncology, pulmonary oncology) |
| --- | --- | --- | --- |
| Length residency program | 2 years | 5 years | 6 years |
| Level of training | 4 in year 1  6 in year 2 | 2 in year 1  2 in year 2  1 in year 3  2 in year 4  1 in year 5 | 1 in year 1  1 in year 4  1 in year 5  1 in year 6 |
| Questionnaires | 10 | 8 | 4 |
| Interviews | 6 | 3 | 3 |
